# The Role of a Cholecystokinin Receptor Antagonist in the Management of Chronic Pancreatitis: A Phase 1 Trial

**DOI:** 10.3390/pharmaceutics16050611

**Published:** 2024-04-30

**Authors:** Victor Ciofoaia, Wenqiang Chen, Bakain W. Tarek, Martha Gay, Narayan Shivapurkar, Jill P. Smith

**Affiliations:** 1 Departments of Gastroenterology and Medicine, MedStar Washington Hospital Center, Washington, DC 20010, USA; victor.ciofoaia@gunet.georgetown.edu (V.C.); tarek.w.bakain@medstar.net (B.W.T.); 2 Department of Medicine, Georgetown University, Washington, DC 20007, USA; wc594@georgetown.edu (W.C.); mdg111@georgetown.edu (M.G.); nms35@georgetown.edu (N.S.)

**Keywords:** chronic pancreatitis, cholecystokinin receptor, pain management

## Abstract

Chronic pancreatitis (CP) is a rare but debilitating condition with an 8-fold increased risk of developing pancreatic cancer. In addition to the symptoms that come from the loss of endocrine and exocrine function in CP, the management of chronic pain is problematic. We previously showed that the CCK-receptor antagonist called proglumide could decrease inflammation, acinar-ductal metaplasia, and fibrosis in murine models of CP. We hypothesized that proglumide would be safe and diminish pain caused by CP. A Phase 1 open-labeled safety study was performed in subjects with clinical and radiographic evidence of CP with moderate to severe pain. After a 4-week observation period, the subjects were treated with proglumide in 400 mg capsules three times daily (1200 mg per day) by mouth for 12 weeks, and then subjects returned for a safety visit 4 weeks after the discontinuation of the study medication. The results of three pain surveys (Numeric Rating Scale, COMPAT-SF, and NIH PROMIS) showed that the patients had significantly less pain after 12 weeks of proglumide compared to the pre-treatment observation phase. Of the eight subjects in this study, two experienced nausea and diarrhea with proglumide. These side effects resolved in one subject with doses reduced to 800 mg per day. No abnormalities were noted in the blood chemistries. A blood microRNA blood biomarker panel that corresponded to pancreatic inflammation and fibrosis showed significant improvement. We conclude that proglumide is safe and well tolerated in most subjects with CP at a dose of 1200 mg per day. Furthermore, proglumide therapy may have a beneficial effect by decreasing pain associated with CP.

## 1. Introduction

Chronic pancreatitis (CP) [1,2] is a syndrome characterized by inflammation and fibrosis of the pancreas resulting in exocrine insufficiency and endocrine dysfunction with decreases in the numbers of acinar and islet cells, respectively [3]. In the past, the etiology of chronic pancreatitis was attributed to chronic alcohol abuse or as a consequence of chronic relapsing idiopathic pancreatitis [4]; however, with recent genetic testing, hereditary causes of chronic pancreatitis have been recognized [5,6]. The most common symptom associated with chronic pancreatitis is abdominal pain, which occurs in up to 80% of affected patients [7]. The treatment of abdominal pain in patients with chronic pancreatitis may consist of combinations of medical, endoscopic, and/or surgical approaches. Unfortunately, these approaches are often not effective, and many of those with chronic pancreatitis are treated with narcotic analgesics. The most serious long-term complication related to chronic pancreatitis, however, is the risk of cancer [8,9]. Chronic pancreatitis is usually diagnosed based on the clinical syndrome (abdominal pain, steatorrhea, and often diabetes), imaging studies, and tests of pancreatic function (fecal elastase) [2]. Endocrine insufficiency is treated with insulin, and exocrine insufficiency is treated with oral pancreatic enzymes to correct malabsorption and prevent diarrhea associated with chronic pancreatitis [10]. Unfortunately, the current treatments only provide temporary pain relief and management of complications and do not arrest or slow the progression of this disabling disease [11]. 

Proglumide is a nonselective cholecystokinin (CCK) receptor antagonist and has unique characteristics that differentiate it from selective CCK-A and CCK-B receptor antagonists [12]. Unlike the other agents, proglumide is water-soluble and can be administered orally. Proglumide was originally developed 30 years ago for peptic ulcer disease [13], and over 600 subjects were studied with dosages of 1200 mg per day without toxicity [13]. However, the commercialization of proglumide was halted with the development of proton pump inhibitors that were much more effective in decreasing gastric acid. In a Phase 1 clinical study in subjects with metabolic syndrome, hepatic steatosis, and fibrosis, three doses were tested, including 800 mg per day, 1200 mg per day, and 1600 mg per day [14]. In this study, both the 1200 mg per day and 1600 mg per day doses were effective and well tolerated; however, the subjects who were taking the higher dose had not yet achieved Cmax proglumide blood levels and steady states by week 12. Hence, the recommendation was to use the 1200 mg per day dose for the current study. Pharmacokinetic studies were also performed with proglumide in healthy controls and in patients with hepatic impairment, which revealed rapid oral absorption and renal excretion [15]. In animal models of chronic pancreatitis, we found that proglumide decreased pancreatic fibrosis and inflammation [16]. Proglumide also decreased the formation of pancreatic acinar ductal metaplasia (ADM) [16] and halted the progression of PanINs (pancreatic intraepithelial neoplasms) [17], thus decreasing the risk of pancreatic cancer. Researchers have previously shown that proglumide could potentiate morphine analgesia following systemic, intrathecal, or intracerebral administration of narcotic analgesic drugs [18]. Another benefit of proglumide is that it has been shown to improve anxiety associated with pain [19]. Proglumide was first recorded to have selective antagonism of excitatory effects of cholecystokinin in the central nervous system in 1983 [20]. Low doses of proglumide induced antinociception in mice [21], and the authors concluded that proglumide may alter morphine resistance.

The purpose of this Phase 1 clinical study was to test the safety of proglumide in subjects with established chronic pancreatitis. The secondary objective was to determine whether proglumide therapy could decrease pain in subjects with chronic pancreatitis and alter the pancreas extracellular matrix using a blood biomarker assay that measures tissue fibrosis and inflammation.

## 2. Materials and Methods

### 2.1. Regulatory and Setting

This prospective open-labeled clinical trial undertaken at Georgetown University was an investigator-initiated translational research study based upon a study on mice that showed reversal inflammation, fibrosis, and acinar ductal metaplasia with proglumide [16]. The research protocol was approved by the FDA under IND#161920 and the Institutional Review Board (Protocol #5063), and the trial was registered at https://classic.clinicaltrials.gov/ct2/show/NCT05551858, accessed on 23 September 2022, with the following registry trial number: NCT#05551858. All participants signed informed consent forms and agreed to participate in this study.

### 2.2. Eligibility Criteria

Subjects with confirmed clinical chronic pancreatitis supported by imaging (CT, MRI, EUS, and/or ERCP) criteria, such as the Cambridge [22] or Rosemont [23] criteria, and with symptoms including pain were eligible if they were between the ages of 18 and 75 years. The inclusion and exclusion criteria are shown in Table 1. Children were not included in this study since proglumide has not been tested in this population. Since pain was a major outcome, subjects were required to have an average pain score of at least 5 on a numeric scale from 0 to 10 to be eligible. Since proglumide is renally excreted, participants were required to have a normal glomerular filtration rate. Female subjects that were pregnant, lactating, or unwilling to avoid pregnancy were ineligible. Likewise, male participants unwilling to use contraception in order to avoid pregnancy were not eligible. Subjects that were actively abusing alcohol or nonprescription drugs could not participate. Because highly potent selective CCK-AR antagonists may impair gall bladder emptying [24], resulting in acute cholecystitis, subjects with active gallbladder disease or gallbladder dyskinesia were not allowed to participate. An advantage of proglumide over the highly selective CCK-A receptor antagonists is that gallbladder dysfunction has never been reported with proglumide.

### 2.3. Study Design and Objectives

**Figure 1:**
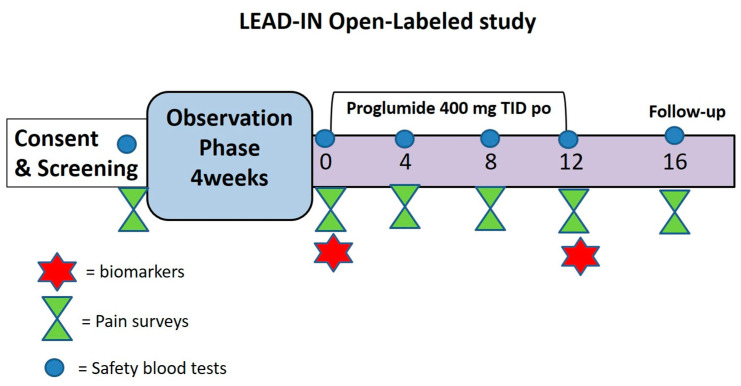
Study design.

The study design of the Phase 1 trial is shown in Figure 1. After screening and meeting the inclusion criteria, eligible subjects entered a 4-week observation period in order to collect information on the frequency and intensity of chronic pancreatitis pain without proglumide. Information regarding pain was collected using a daily symptom diary and completion pain surveys (see below) during the observation phase. Mean scores of pain surveys performed at the screening and baseline visits without proglumide were averaged and compared to the pain scores over the following 4, 8, and 12 weeks when subjects received proglumide. After the baseline visit, subjects had follow-up visits at 4-week intervals, and after 12 weeks of proglumide, a safety follow-up visit occurred 4 weeks after the completion of the study. Adverse events were recorded, and laboratory tests were analyzed every four weeks while on therapy for safety analysis. In addition to the safety blood that was drawn every 4 weeks, an additional blood sample was collected at baseline and after 12 weeks of therapy for a blood biomarker assay for pancreatic fibrosis and inflammation. The objective of the Phase 1 protocol was to assure that the dose of proglumide selected was safe and tolerated in subjects with chronic pancreatitis. The secondary objective was to determine if the pain surveys were feasible, easy to complete, and could be applied to the Phase 2 trial. The third objective was to analyze the blood biomarker panel to determine if proglumide therapy resulted in a change in microRNAs associated with decreased tissue fibrosis and inflammation.

### 2.4. Intervention

Proglumide was manufactured by an FDA-approved facility, COSMA S.p.A. (Milan, Italy). The drug was compounded into 400 mg vegan capsules by Custom Scripts (Lancaster, PA, USA). Proglumide was dispensed from the investigational pharmacy at baseline and at the week 4 and week 8 visits and prescribed as one capsule by mouth three times daily for a total daily dose of 1200 mg. The dose and dosing schedule were based upon the results of a prior study involving subjects with metabolic-associated hepatic steatosis [14] and a pharmacokinetic study involving healthy controls and subjects with hepatic impairment [15]. The protocol allowed for the dose to be reduced from 1200 mg/day to 800 mg/day for drug-related side effects.

### 2.5. Study Assessments

#### 2.5.1. Safety

The primary goal of this Phase 1 study was safety, and this parameter was assessed by comparing the blood tests at screening and baseline with the blood tests with proglumide use during the 12-week treatment period. Blood tests included basic chemistry with renal profile, complete blood count (CBC) and platelets, serum lipase levels, HbA1C (glycosylated hemoglobin), and C-reactive protein (CRP). Safety was evaluated using the Common Terminology Criteria for Adverse Events (CTCAE) version 5 [25]. Safety was also evaluated based on the number of adverse events reported. Another objective of the protocol was to assure the dose of proglumide selected was tolerated in subjects with chronic pancreatitis. If the 1200 mg daily dose of proglumide caused side effects that did not resolve spontaneously, the drug could be held for 7–14 days. If the side effect resolved in this time, the protocol called for proglumide to be restarted at a lower dose of 800 mg/day. If the side effects returned on the lower dose of proglumide, the drug was stopped, and the subject was withdrawn from the study.

#### 2.5.2. Pain Evaluation

Pain can be difficult to assess because it is subjective, and for that reason, we used multiple outcomes. There are multiple measures available to assess pain in adult populations, and several different scoring measures have been reviewed [26]. Each measure has its own strengths and weaknesses. The first pain survey we utilized was the Numeric Rating Scale. Both the Visual Analog Scale for Pain and the Numeric Rating Scale (NRS) for pain are unidimensional single-item scales that provide an estimate of patients’ pain intensity. They are easy to administer, complete, and score. Of the two, the pain NRS is preferred at the point of patient care due to simpler scoring. The NRS is a segmented numeric version of the visual analog scale (VAS) in which a respondent selects a whole number (0–10 integers) that best reflects the intensity of their pain [27]. The common format is a horizontal bar or line [28]. The NRS survey was the primary pain survey used in this Phase 1 trial, and it was completed by the subjects for the 7 days prior to each visit, and they were instructed to record the score of the worse pain for a 24 h period.

We utilized the PROMIS instruments to measure aspects of pain for the second pain survey. PROMIS was first developed as an initiative at NIH (PROMIS; www.nihpromis.org, accessed on 1 March 2024) to provide item banks that offer the potential for efficient, flexible, and precise measurement of commonly studied patient-reported outcomes [29]. PROMIS instruments use modern measurement theory to assess patient-reported health statuses for physical, mental, and social well-being to reliably and validly measure patient-reported outcomes (PROs) for clinical research and practice. PROMIS instruments are generated from item banks. 

The third pain survey we used was the Comprehensive Pain Assessment Tool for Chronic Pancreatitis, or COMPAT-SF in short form. The COMPAT-SF [30] includes general aspects of pain and features that separate patients with chronic pancreatitis from other patients with chronic pain. The COMPAT-SF can be completed within 10 min, and it was validated and tested for reliability. The developed short form (SF) comprises 5 pain dimensions and includes 6 questions.

Subjects were asked to keep a record of other pain medications used, such as aspirin, acetaminophen, and NSAIDs, on a daily basis. They carried this out during the observation phase and then continuously throughout the study. If the subjects were taking narcotic analgesics for pain management, these were permitted and continued during this trial.

#### 2.5.3. Biomarker Panel

A noninvasive blood microRNA (miRNA) biomarker panel for pancreatic fibrosis and inflammation was analyzed at baseline and again at week 12. RNA was extracted from serum samples from patients with chronic pancreatitis using miRNeasy serum plasma kit (Qiagen, Germantown, MD, USA). miRNA was converted to cDNA using the miRCURY SYBR Green RT Kit (Qiagen). miRNA expression profiling was performed using miRCURY SYBR green PCR master mixture (Qiagen) containing QuantiNova DNA polymerase and SYBRGreen and miRNA specific primers for the above miRNAs. The detection of a fluorescent signal due to the amplification of cDNA was carried out in an ABI 7500 Real-Time PCR system (Applied Biosystems, Thermo Fisher, Waltham, MA, USA). Cycle threshold (Ct) data were collected, and the relative difference between the two groups was calculated using the ∆∆Ct method. The most stably expressed microRNA (miR-16-5p) was used as the normalizer. A dissociation curve analysis of the PCR products was performed to confirm the specificity of amplification (Supplemental Figure S1). We used several miRNAs that are involved in fibrosis and/or inflammation that are detected in the blood and correlate with histology. Serum levels of three selective miRNAs that have been shown to inhibit tissue fibrosis (e.g., miR-185-5p, miR-378a-3p, and miR-346-5p) were determined [31,32,33,34], and these were measured in the subjects’ blood. Another miRNA, miR-122-5p, has been found to be elevated in subjects with pancreatic inflammation and decreases with less inflammation [35]. MiR-122 is also increased in pancreatic cancer [36].

### 2.6. Statistics

Data analysis for this study was conducted using SPSS version 18.0. Initially, descriptive statistics were utilized to summarize the sample characteristics and main variables derived from the Numeric Rating Scale, COMPAT-SF, and PROMIS assessments and patients’ laboratory results. These descriptive analyses included mean, median, standard deviation, and range to provide a comprehensive overview of the data collected. For continuous variables from certain scores within the Numeric Rating Scale, COMPAT-SF, PROMIS, and lab results, t-tests, one-way ANOVA, or Fisher’s exact test were used to compare differences between groups. All data were assessed with intent-to-treat analysis. All tests were two-tailed, and a *p* value less than 0.05 was considered to be statistically significant.

## 3. Results

### 3.1. Study Participants

Nine subjects were screened, and one did not meet the eligibility criteria. Eight subjects who met the eligibility criteria with clinical and radiographic evidence of chronic pancreatitis and moderate to severe pain were enrolled at Georgetown University Medical Center between 12 December 2022 and 15 December 2023. The demographics of the subjects screened for the study are shown in Table 2. Two of the nine subjects were females, and the rest were males. Male predominance is typical of chronic pancreatitis [4]. Two subjects were black, and the rest were Caucasian. Subject 3 was a screen failure and was not treated with proglumide. Of the eight eligible subjects treated with proglumide, the average age was 47.6 ± 5.3 years (range 21–68 years). The majority of subjects in this clinical trial had a genetic alteration as the etiology of their chronic pancreatitis (Table 2). One subject required morphine for pain control, and a second subject was taking extended-release oxycodone. The other participants were not taking narcotic analgesics but were using acetaminophen as needed for pain.

### 3.2. Safety and Laboratory Testing

There were no serious adverse events reported, and the blood hematology and chemistry tests did not change significantly over the 12-week period compared to the baseline values. One subject noted continued pancreatitis symptoms with abdominal pain, nausea, and diarrhea that did not improve, and the subject prematurely discontinued the trial in month 3. Another subject reported nausea, insomnia, and leg cramps while taking proglumide at a dose of 1200 mg per day, but the side effects resolved with a dose reduction to 800 mg per day, and the subject completed the trial.

The values (mean ± SEM) of the laboratory tests performed at the baseline visit and the week-12 visit are shown in Table 3 with normal ranges. The only chemistry value that increased at week 12 was serum chloride. A defective cystic fibrosis transmembrane conductance regulator (CFTR) protein can lead to excessive losses of sodium and chloride in sweat, which results in hypochloremia and hyponatremia [37]. Although CFTR mutations were only reported in three of our eight subjects, these three patients exhibited the greatest increases in serum chloride at week 12. The mean serum sodium values also increased over the 12-week period, suggesting that proglumide was improving the function of the CFTR. The mean baseline aspartate transaminase (AST) liver enzyme value was slightly above the normal range. This elevation was attributed to an abnormal value in subject 6 that had known fatty liver disease. This subject’s liver AST improved with proglumide therapy. We previously reported that proglumide decreases inflammation and hepatic transaminase levels in nonalcoholic fatty liver disease [14]. The C-reactive protein (CRP) values decreased compared to the baseline values during the course of the study, suggesting decreased inflammation, but this difference was not significant.

### 3.3. Pain Surveys

**Figure 2:**
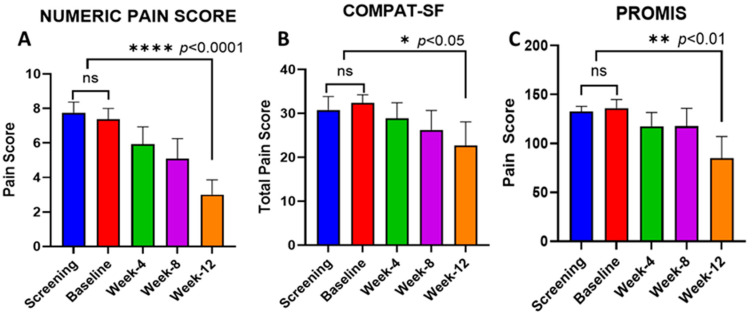
Results of pain surveys completed by subjects with chronic pancreatitis compared to baseline pain scores; there was significant improvement in pain at week 12 of study as measured with (**A**) Numeric Rating Scale, (**B**) COMPAT-SF, and (**C**) NIH PROMIS. Columns represent means ± SEM with 8 samples per column. ns = not significant; * *p* < 0.05; **p < 0.01; and **** *p* < 0.0001.

Three pain surveys were performed at the screening, baseline, week-4, week-8, and week-12 visits. Using all three pain surveys, we did not observe any change in the pain scores over the 4-week observation period before the initiation of proglumide (screening to baseline visits). The results of the surveys before proglumide therapy (screening and baseline) were compared to the pain survey results obtained while patients were taking proglumide. Compared to the pre-treatment pain scores, the pain scores at week 12 of proglumide therapy were significantly improved based on all three of the pain surveys administered in this study. Although the pain scores decreased slightly at weeks 4 and 8, these changes were not significant. The results of the pain surveys from each visit are shown in Figure 2. The primary pain survey, the Numeric Rating Scale, demonstrated the greatest change at week 12 from the pretreatment values with a decrease of 61% in pain scores. The other two pain surveys demonstrated significant improvements in the pain scores after 12 weeks, with the COMPAT-SF scores decreasing by 30% and the PROMIS survey scores decreasing by 37.5% compared to the baseline values. The pain surveys were not cumbersome, and the subjects were able to easily complete them without difficulty.

### 3.4. Blood Biomarker Assay

**Figure 3:**
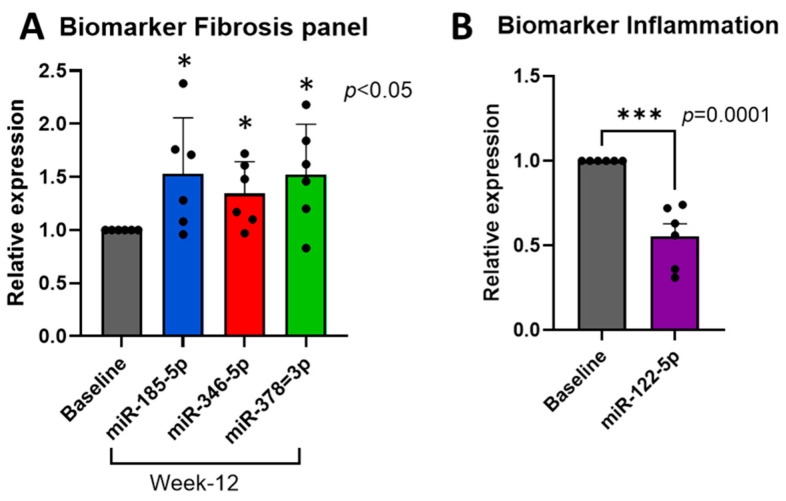
Blood biomarker panel for chronic pancreatitis. (**A**) miRNA expression of biomarkers indicative of decreasing fibrosis is increased in subjects after 12 weeks of proglumide compared to baseline. (**B**) Decrease in miR-122-5p compared to baseline values correlates with less inflammation and decreased risk of pancreatic cancer. Columns represent means ± SEM with 6 samples per column. * *p* < 0.05; *** *p* = 0.0001. Black dots represent individual values.

Since miRNAs are stable in the peripheral blood, we evaluated our panel of miRNA biomarkers as a noninvasive test to study changes in the pancreas parenchyma after 12 weeks of proglumide therapy compared to the pretreatment values. The microRNAs (miR) miR-185-5p, miR-346-5p, and miR-378-3p are known to inhibit fibrosis from stellate cells and cancer-associated fibroblasts [31,33,34,38]. Two subjects did not have either pre- or post-treatment biomarker samples drawn, and because we were comparing the change in the miRNA levels, only six matched samples were available for the biomarker analysis. In our analyses of the serum samples from the available patients with chronic pancreatitis, we found a statistically significant increase in the serum miRNA levels of miR-185-5p (1 ± 0 to 1.53 ± 0.22), miR-346-5p (1 ± 0 to 1.34 ± 0.12), and miR-378-3p (1 ± 0 to 1.52 ± 0.19) from the baseline to week 12 of the proglumide treatment (Figure 3A). This miRNA blood biomarker is consistent with the inhibition of fibrosis (or increased fibrinolysis). We found a statistically significant decrease in the serum miRNA levels of miR-122-5p (1 ± 0 to 0.55 ± 0.0.074) from the baseline to week 12 of the proglumide treatment (Figure 3B). This decrease in miR-122-5p is indicative of less inflammation [35] and a decreased risk of pancreatic cancer [36] (Figure 3B). Detailed Ct data and relative expression data are shown in Supplemental Table S1.

## 4. Discussion

This study represents a novel approach to treat chronic pancreatitis using an oral CCK receptor antagonist. This Phase 1 trial met its primary objective of achieving safety, and proglumide was found to have a broad safety profile in subjects with chronic pancreatitis. We reported important changes in pain surveys and a blood biomarker panel that was consistent with clinical improvement in symptoms and the pancreas microenvironment in the duration of this study. The significant improvement in these parameters occurred during the 12-week duration of this study, suggesting that this length of time will be adequate to evaluate efficacy in a Phase 2 clinical trial. The starting dose of 1200 mg daily in this study was tolerated by most individuals with chronic pancreatitis; therefore, the optimal starting dose for the Phase 2 trial appears to be 1200 mg per day.

This study demonstrated, for the first time, that the oral administration of proglumide monotherapy is effective in decreasing pain even in the absence of narcotic analgesics. Some studies have shown that proglumide may potentiate morphine analgesia [18]; however, the majority of the subjects in the current study were not taking narcotics. Only two of the eight subjects in this study were taking narcotic analgesics upon enrollment, and one was able to discontinue narcotic use without an exacerbation of abdominal pain. Therefore, the analgesic effect discovered with the use of proglumide in the majority of the subjects with chronic pancreatitis was not attributed to the potentiation of morphine. The decrease in pain in our subjects did not occur instantly, but there was a significant reduction in pain over several weeks of oral proglumide therapy. There are several potential mechanisms through which proglumide may decrease pain in pancreatitis. Cholecystokinin (CCK) is a peptide that is widely distributed throughout the brain, where it possesses properties of a neurotransmitter. Two receptor subtypes have been identified to date—the CCK-A and CCK-B receptors (also referred to as CCK-1R and CCK-2R). The distribution of the CCK-B subtype is widespread in the brain [39,40]. The CCK-A subtype is predominant in the peripheral nervous system [41,42]. One mechanism that may be involved with the decrease in pain could be the selective antagonism of the excitatory effects of cholecystokinin in the central nervous system [20] or the decreased inflammation of the nerves in the peri-pancreatic bed surrounding the pancreas. G-protein-coupled receptors (GPCRs) are known to “cross-talk” or influence the action of other GPCRs, either by sensitizing or desensitizing the intracellular signaling or downstream pathways of each other or by forming heterodimers [43] to mediate physiologic effects. CCK receptors and opioid receptors are both in the family of GPCRs; therefore, the receptor blockade of the CCK receptor with proglumide may desensitize opioid receptors and lessen pain perception.

Another class of pain opioid-receptor-like (ORL1) proteins has been identified [44]. This new receptor shows an overall 60% homology with the classic opioid receptors [45]. The nociceptin/orphanin FQ (NC) and its receptor (NOP) represent a novel peptide/receptor system that is pharmacologically distinct from classical opioid systems [44]. Because nociceptin receptors are located in the pain processing pathways in the dorsal root ganglia, spinal dorsal horn, and brain, several researchers have investigated the roles of the central NOP receptor system in pain modulation [46]. Unlike the classic mu-opioid receptor agonists, such as morphine, NOP receptor agonists do not have undesirable adverse effects in non-human primates, and more importantly, they synergistically enhance the classic mu-opioid receptor agonist-induced analgesia [46]. New compounds are being developed that are agonists at the NOP receptors and potentiate analgesia [47]. Drugs that serve as agonists at the NOP receptor are structurally of the benzenoid class and are being developed to be used for pain management [48]. Similar to the benzenoid aromatic compounds that bind to the NOP receptor, proglumide also has a benzene ring [49], making it a potential candidate for interaction with the NOP receptor.

Pancreatic stellate cells (PSCs) are resident pancreatic fibroblasts that play important roles in chronic pancreatitis and fibrosis [50]. PSCs are quiescent and regulate the extracellular matrix (ECM) and homeostasis. When PSCs are activated during injury or with inflammation (pancreatitis), they express α-SMA (smooth muscle actin) and produce proteins (collagen I, collagen III, and fibronectin) [51], resulting in the fibrosis characteristic of chronic pancreatitis [52,53].

Both CCK-A and CCK-B receptors have been found on pancreatic stellate cells [54], and when activated, they deposit collagen. The fibrosis and desmoplastic microenvironments found in those with chronic pancreatitis are also characteristic of pancreatic cancer [55]. Using cultured murine and human pancreatic stellate cells in vitro, we previously demonstrated that proglumide induced the plasticity of PSCs, rendering them quiescent with decreased migration, proliferation, and collagen formation [56]. Proglumide exhibited unique anti-fibrotic properties in that, unlike the other CCK receptor antagonists, proglumide decreased pro-collagen1α1 and mature collagen protein expression, and it also increased collagen cleavage/degradation products due to the breakdown of fibrosis. These anti-fibrotic characteristics elicited by proglumide may occur through a non-receptor-mediated mechanism.

Drug development is a costly and timely business, and it can take decades of research time and millions of dollars before a compound is approved by the FDA for therapy. One strategy to decrease the cost and shorten the time interval for therapies is to re-purpose older drugs that have already been tested in animals and humans. This Phase 1 study showed that proglumide has a safe profile in subjects with chronic pancreatitis and has the potential to not only control the symptoms of chronic pancreatitis, but also to possibly restore pancreatic architecture and function.

## 5. Conclusions

Currently, there are only symptomatic treatments available for subjects with chronic pancreatitis. None of these ‘supportive’ medications help to improve pancreatic function or reverse the underlying damage and decrease the risk of cancer. We have shown that proglumide improves histology and function in chronic pancreatitis in animal models. A very important aspect of proglumide therapy that will potentially benefit subjects with chronic pancreatitis is proglumide’s analgesic effect and improvement of pain. Proglumide also decreases acinar ductal metaplasia and PanIN progression [16], and hence, it may lower the risk of developing pancreatic cancer. The idea of using the same oral drug to treat the clinical symptoms associated with chronic pancreatitis and reverse the histological damage of the pancreas and prevent further damage is indeed novel.

## 6. Patents

Georgetown University owns a patent concerning this work under US patent No. 11,278,551 issued on 22 March 2022, and Dr. Jill Smith is listed as an inventor.

## Figures and Tables

**Table 1:** Eligibility criteria.

Inclusion Criteria	Exclusion Criteria
Males or females aged 18 to 75 years Clinical symptoms of chronic pancreatitis Confirmation of chronic pancreatitis with imaging (Cambridge classification), EUS, biopsy, or fecal elastase < 200 µg/g, or abnormal 72 h fecal fat and radiographic evidence of CP Pain not adequately controlled with medications Pain score of at least 5 on a numeric pain scale of 0–10 Use of stable doses of anti-diabetic medication for at least 90 days prior to screening Female subjects of reproductive potential and male subjects with female partners of reproductive potential who agree to use acceptable contraception and/or true abstinence prior to study entry for the duration of study participation and up to 3 months after stopping therapy; male subjects with partners who are currently pregnant who agree to condom use during intercourse upon study entry for the duration of study participation and up to 3 months after stopping therapy Both males and females must be willing and able to continue contraception to prevent pregnancy for 3 months after the completion of the study	Currently abusing alcohol (more than three drinks in a day or more than seven drinks per week) or nonprescription drugs Pregnant, lactating, or unwilling to prevent pregnancy Renal insufficiency; CKD; GFR < 60 mL/min Unable to sign consent form or maintain a diary Liver enzymes > 2× ULN, Hgb ≤ 8.5, Creat ≥ 2; HgbA1c > 8 Type 1 diabetes Subjects with confirmed cirrhosis Evidence of active gallbladder disease or gallbladder dyskinesia Female subjects of reproductive potential and male subjects with female partners of reproductive potential who are unwilling to use acceptable contraception and/or true abstinence prior to study entry for the duration of study participation and up to 3 months after stopping therapy Male subjects with partners who are currently pregnant that are unwilling to use condoms during intercourse prior to study entry for the duration of study participation and up to 3 months after stopping therapy.

**Table 2:** Participant demographics.

Subject Number	Age (years)	Sex	Race	Etiology
PROG1001	61	M	W	CFTR
PROG1002	38	F	W	SPINK1
PROG1003 *	29	F	B	SPINK1
PROG1004	21	M	W	CFTR
PROG1005	52	M	W	SPINK1
PROG1006	45	M	B	Autoimmune
PROG1007	56	M	W	Hyperlipidemia
PROG1008	68	M	W	Alcohol
PROG1009	40	M	W	CFTR, CLDN2

M = male; F = female; W = white or Caucasian; B = black; CFTR = cystic fibrosis transmembrane conductance regulator gene; SPINK1 = Serine Peptidase Inhibitor Kazal Type 1 gene; CLDN2 = claudin 2 gene. * denotes screening failure; subject was not treated.

**Table 3:** Laboratory blood tests before and after proglumide therapy.

Test/(Units)	Normal Range	Baseline	Week 12	*p* Value
WBC (k/µL)	4.0–10.8	6.8 ± 0.78	7.1 ± 1.3	0.828
Hgb (g/dL)	12.5–16.5	14.0 ± 0.62	13.8 ± 0.76	0.215
Platelet (k/µL)	145–400	256 ± 16	246 ± 23	0.727
Lipase (units/L)	2–53	36.8 ± 3.5	34.3 ± 3.2	0.607
Sodium (mmol/L)	136–145	139.6 ± 0.95	141.3 ± 1.6	0.168
Potassium (mmol/L)	3.4–4.5	4.26 ± 0.12	4.18 ± 0.20	0.745
Chloride (mmol/L)	98–107	103 ± 2.8	108 ± 3.0	0.013 *
CO_2_ (mmol/L)	20–31	26.6 ± 0.6	25.8 ± 1.8	0.719
BUN (mg/dL)	9–23	13 ± 0.72	14 ± 0.86	0.391
Creatinine (mg/dL)	0.6–1.1	0.95 ± 0.04	0.96 ± 0.05	0.858
Calcium (mg/dL)	8.7–10.4	9.5 ± 0.1	9.5 ± 0.2	0.885
Total Bilirubin (mg/dL)	0.3–1.2	0.51 ± 0.08	0.58 ± 0.13	0.646
AST (U/L)	0–33	38.3 ± 14.6 #	23 ± 2.7	0.334
ALT (U/L)	0–49	38 ± 18.8	20.8 ± 4.8	0.403
Alkaline Phosphatase (U/L)	46–116	79.8 ± 11.7	75.8 ± 12.7	0.822
Albumin (g/dL)	3.2–4.8	4.57 ± 0.06	4.63 ± 0.11	0.653
Total Protein (g/dL)	5.7–8.2	7.4 ± 0.10	7.4 ± 0.14	0.972
HgbA1C (%)	3.8–5.6	5.33 ± 0.16	5.45 ± 0.16	0.581
CRP (mg/L)	0–10	5.6 ± 1.4	4.16 ± 1.17	0.457

Data represent means ± SEM with N = 8 samples per analyzed test. * represents a value that is statistically significant. # represents a valued that was greater than the upper limit of normal.

## Data Availability

The data will be available on the clinicaltrials.gov (accessed on 1 March 2024) website when the study is completed.

## Supplementary Materials

The following supporting information can be downloaded at https://www.mdpi.com/article/10.3390/pharmaceutics16050611/s1, Supplemental Figure S1: Representative dissociation curve analysis of qPCR amplification for different microRNAs using total RNA from serum samples from patients. Supplemental Table S1: Raw Ct data and calculation of relative expression data from raw Ct data.
