# Supplementary material for: The Role of a Cholecystokinin Receptor Antagonist in the Management of Chronic Pancreatitis: A Phase 1 Trial
Source: Pharmaceutics. 2024 Apr 30;16(5):611. doi: 10.3390/pharmaceutics16050611 (PMC11125239; doi:10.3390/pharmaceutics16050611)
Supplement: Supplementary file 1 [file pharmaceutics-16-00611-s001.zip › pharmaceutics-2921022-supplementary.pdf]

Supplemental Data:

Role of a Cholecystokinin Receptor Antagonist in the Management of Chronic Pancreatitis: A Phase 1 Trial

Supplemental Figure S1: Representative dissociation curve analysis of qPCR amplification for different microRNAs using total RNA from serum samples from patients.

Supplemental Table S1: Raw Ct Data and calculation of Relative expression data from the raw Ct data

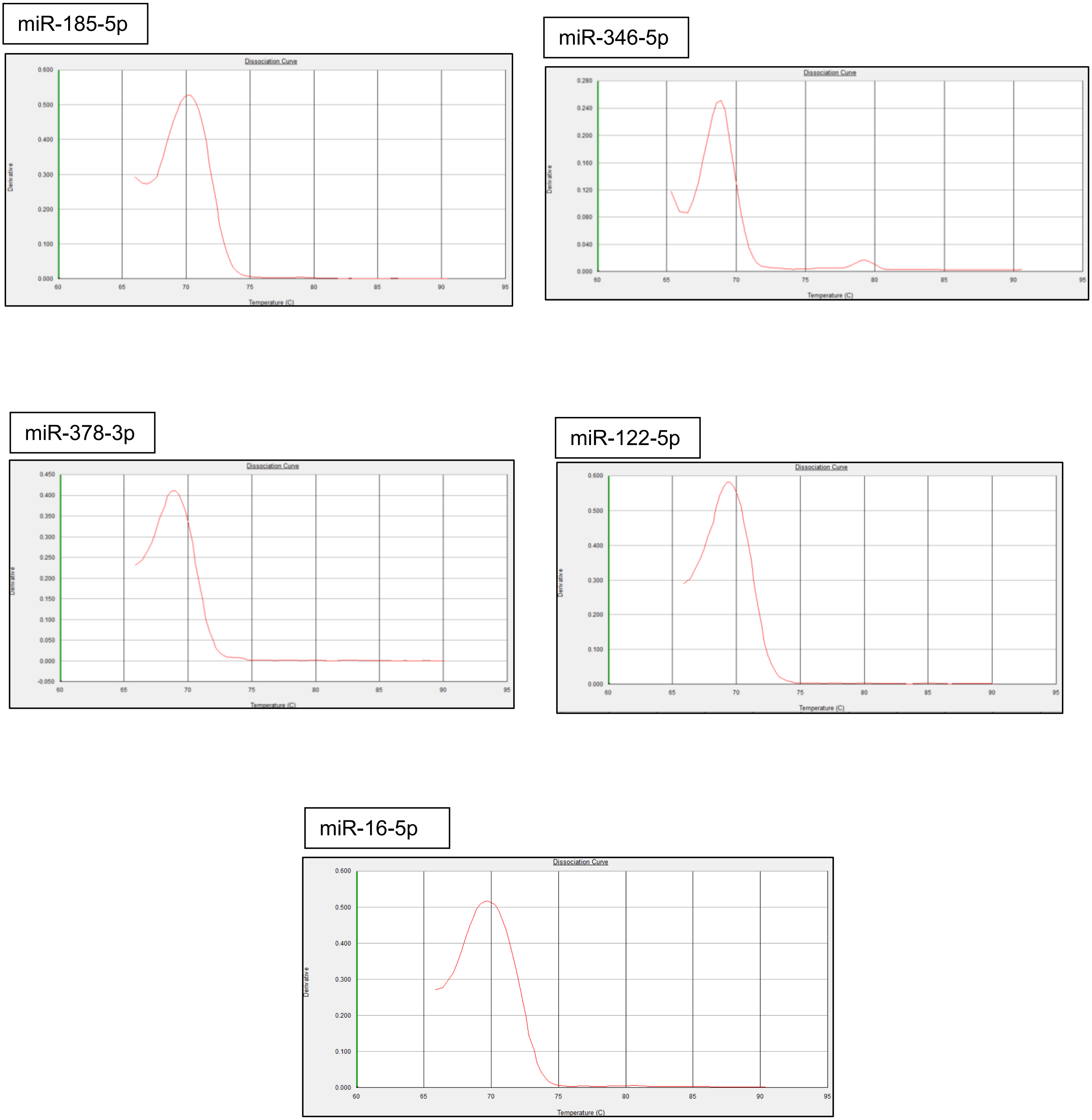

Supplemental Figure S1. Representative dissociation curve analysis of qPCR amplification for different microRNAs using total RNA from serum samples from patients. The figure shows single peak in the dissociation curve analysis in each case indicative of single amplicon formed following PCR amplification (specificity) in case of all genes.

Supplemental Table S1: Raw Ct Data and calculation of Relative expression data from the raw Ct data												
	**Ct values of microRNAs in serum samples at baseline level (0) compared to at 12 wks treatment of chronic pancreatitis patients with proglumide**											
**microRNA**	001-0	001-12	002-0	002-12	004-0	004-12	007-0	007-12	008-0	008-12	009-0	009-12
**miR-185-5p**	27.2155	25.4207	23.1836	25.4265	23.7198	23.6912	30.5318	29.4638	30.8696	26.9163	29.1124	25.1734
**miR-346-5p**	34.0967	33.1237	32.5902	33.9895	34.2977	34.0501	35	33.3435	33.8161	30.8934	32.7829	29.5333
**miR-378-3p**	31.0512	29.152	27.0553	29.4993	28.0594	27.8252	33.022	31.837	33.1859	29.7854	33.6097	29.3584
**miR-122-5p**	28.7648	28.4023	26.1191	29.1443	25.7463	26.545	30.246	30.7687	29.424	28.4026	28.436	25.7401
**miR-16-5p**	22.6333	21.6134	18.6272	20.8052	19.1048	19.429	25.9367	24.9688	25.0683	22.3678	24.417	21.2932
	**Fold change (Relative expression) Fold Change (Relative expression) data were calculated with ΔΔCT method using raw Ct data as described in Materials and Methods)**											
	**Relative expression of microRNAs in serum samples at baseline level (0) compared to at 12 wks treatment of chronic pancreatitis patients with proglumide**											
**microRNA**	001-0	001-12	002-0	002-12	004-0	004-12	007-0	007-12	008-0	008-12	009-0	009-12
**miR-185-5p**	1	1.71	1	0.96	1	1.28	1	1.08	1	2.38	1	1.76
**miR-346-5p**	1	0.97	1	1.72	1	1.48	1	1.61	1	1.17	1	1.1
**miR-378-3p**	1	1.84	1	0.83	1	1.46	1	1.2	1	1.62	1	2.18
**miR-122-5p**	1	0.63	1	0.56	1	0.72	1	0.36	1	0.31	1	0.74
	miR-16-5p most stably expressed microRNA was used as normalizer											

Effect of Proglumide administration on microRNA expression in serum of Chronic pancreatitis patients

	**0 wk**	**12 wk**		
**microRNA**	**Mean ±Std Error**	**Mean ±Std Error**	**p value**	
miR-185-5p	1 ± 0	1.53 ± 0.22	0.03	*
miR-346-5p	1 ± 0	1.34 ± 0.12	0.02	*
miR-378-3p	1 ± 0	1.52 ± 0.19	0.02	*
miR-122-5p	1 ± 0	0.55 ± 0.074	0.0001	*

*=statistically significant

miRNA expression was quantitated using qPCR analysis

miR-16-5p most stably expressed microRNA was used as normalizer
